# Spatial networks differ when food supply changes: Foraging strategy of Egyptian fruit bats

**DOI:** 10.1371/journal.pone.0229110

**Published:** 2020-02-25

**Authors:** Erik Bachorec, Ivan Horáček, Pavel Hulva, Adam Konečný, Radek K. Lučan, Petr Jedlička, Wael M. Shohdi, Šimon Řeřucha, Mounir Abi-Said, Tomáš Bartonička

**Affiliations:** 1 Department of Botany and Zoology, Masaryk University, Kotlářská, Brno, Czech Republic; 2 Department of Zoology, Charles University in Prague, Viničná, Prague, Czech Republic; 3 Institute of Scientific Instruments of the Czech Academy of Sciences (ISI), Královopolská, Brno, Czech Republic; 4 Nature Conservation Egypt, Mohandeseen, Giza, Egypt; 5 Department of Earth and Life Sciences, Faculty of Sciences II, Lebanese University, Fanar, Lebanon; Kyoto University, JAPAN

## Abstract

Animals are faced with a range of ecological constraints that shape their behavioural decisions. Habitat features that affect resource abundance will also have an impact, especially as regards spatial distribution, which will in turn affect associations between the animals. Here we utilised a network approach, using spatial and genetic data, to describe patterns in use of space (foraging sites) by free-ranging Egyptian fruit bats (*Rousettus aegyptiacus*) at the Dakhla Oasis in Egypt. We observed a decrease in home range size during spring, when food availability was lowest, which was reflected by differences in space sharing networks. Our data showed that when food was abundant, space sharing networks were less connected and more related individuals shared more foraging sites. In comparison, when food was scarce the bats had few possibilities to decide where and with whom to forage. Overall, both networks had high mean degree, suggesting communal knowledge of predictable food distribution.

## Introduction

Behaviour is expressed as a response to intrinsic and extrinsic factors that include an individual’s physical and social environment, the latter comprising non-random and heterogeneous social interactions [1]. This response generally occurs in two major forms, i.e. innate behaviour and learned behaviour. Instinct-driven behaviour (i.e. innate behaviour) is genetically incorporated as soft wired “tendencies” that are vertically inherited from parents to offspring. These can be moulded by lifetime experiences (i.e. learned behaviour), allowing an individual to acquire knowledge about the environment and adapt to it [2–5]. Learned behaviour, the product of an animal’s contact with the environment, is acquired by either individual or social learning and can spread both vertically and horizontally among individuals [6]. A key challenge in understanding such differences in behaviour is to disentangle the contribution of individual experience and social learning (mediated through cultural transmission of knowledge) from innate programs (inherited genetically) [7].

Group-living animals, especially those that rely on seasonally fluctuating resources (e.g. bats), benefit from an ability to utilise social information [8]. In this way, social interactions facilitate information gathering and learning from another individual’s behaviour or its products [9, 10]. In the context of foraging, social information is used by naive individuals to locate resources and is acquired by observing the behaviour of successful foragers [11], whereby inadvertent cues produced by feeding conspecifics or heterospecifics may attract individuals to the resource. Such local enhancement has been observed in a range of taxa such as crustaceans (*Paguroidea* spp., [11]), fish (*Poecilia reticulata*, [12]) or mammals (*Sus scrofa*, [13]; *Heterocephalus glaber*, [14]; *Phyllostomus hastatus*, [15]). By observing the behaviour of conspecifics, individuals may also acquire information on roosting sites, the edibility of new food sources or new skills [16].

Interaction between individuals requires relatively close spatial proximity, with the social environment mainly determined by their movement decisions [17–20]. Habitat features, such as resources, play a fundamental role in the spatial distribution of group members, shaping inter-individual proximity and potential social interactions [18, 21–24]. In social mammals, group members tend to live together with relatives, and thus have more opportunities for social learning from related individuals [25]. In this way, genetic relatedness between individuals may also play a role in the socio-spatial organisation of populations [26]. Kin-biased associations can result in numerous fitness benefits including increased foraging efficiency [27–30]. In two bat species, i.e. Bechstein’s bat (*Myotis bechsteinii*) [31, 32] and the greater horseshoe bat (*Rhinolophus ferrumequinum*) [33], related individuals learn where and what to eat, share space (e.g. foraging sites) and/or forage socially. A recent study, however, found that while frugivorous bat species (i.e. *Rousettus*) have not been observed searching for food in groups, yet groups have been observed at foraging sites interacting with each other [34].

Here, we report on variation and seasonal changes in spatial activity and space sharing by Egyptian fruit bats (*Rousettus aegyptiacus*, [35]), a medium-sized (100–200 g) pteropodid bat with a large polytopic distributional range encompassing several climatic zones, including the tropics, subtropics and temperate regions such as the eastern Mediterranean [36]. Egyptian fruit bats roost in caves or artificial structures (e.g. abandoned buildings, tombs and mines) and form colonies ranging from just a few individuals to several thousands. Within the roost, bats maintain close body contact with other individuals and interact with each other [37]. Proportion of pregnant females occurs in two peaks, in late spring and in autumn. Reproductive activity of males is the highest during autumn and winter months (more details in [38]). The diet of these fruit bats consists mainly of fruit, such as figs (*Ficus carica*, *F*. *microcarpa*, *F*. *religiosa*, *F*. *rubiginosa*), dates (*Phoenix dactylifera*), loquats (*Eriobotrya japonica*) and mulberries (*Morus nigra*) [39, 40]. Strong seasonality in the Eastern Mediterranean climate results in an uneven distribution of food sources, though, conditions are even harsher in desert environments such as the Dakhla oasis [41, 42]. Owing to a lack of rainfall and limited groundwater resources, summer is the only season when food is plentiful, the rest of the year being characterized by food scarcity. This is further exacerbated by the harvesting of cultivated crops, especially dates. Under such harsh conditions, a principal challenge in understanding the behaviour of fruit bats is identifying exactly how space-use and inter-individual associations translate into foraging performance.

This paper explores a possible link between genetic relatedness, space sharing and the role of food availability in shaping associations among Egyptian fruit bats. The social network approach [43, 44], which has previously been applied to bats, provides a suitable method for studying these processes [45–49]. Because grouping with kin is believed to reduce some foraging costs and having more relatives could result in easier access to information about foraging sites [33, 50, 51], we predicted a positive association between genetic relatedness and foraging site sharing. As climatic conditions and harvesting activities affect food distribution, we also hypothesised that in spring, when food offer is scarce, animals will share sites where food is still available and therefore the network will be more interconnected. On the other hand, when food offer is rich animals will be more spatially distributed and the group cohesion will decrease.

## Materials and methods

### Study site

All fieldwork took place at the Dakhla oasis in central Egypt (25.6949453N, 28.8831228E) and covered three distinct seasons: winter (November—December 2010), spring (March—April 2011) and summer (July—August 2011). The surrounding environment is typified by houses with gardens, date palm plantations and patches of fig and mango trees. As with much of Egypt, the Dakhla oasis has a hot-desert climate [52], with a mean winter (January) and spring (April) temperature of 19°C, a mean summer (August) temperature of 23°C and mean rainfall of 2 mm in winter, 1 mm in spring and 0 mm in summer. The Egyptian fruit bat population at the Dakhla oasis consists of approximately 2500 individuals in 25 colonies [38]. The colony occupying the Al Qasr old town consisted approximately of 1000 individuals which used 21 different roosts.

### Sampling and radiotracking

Fruit bats were mist netted at foraging sites, whereupon they were weighed and sexed, and a 3.0 mm circular piece of tissue was taken from the wing membrane of for genetic analysis, using a sterile biopsy punch (Miltex, Inc., USA). Age estimation was based on the forearm growth curve published by Mutere [53], dentition, testes size and position in males and the state of nipples in females [38, 40]. The fruit bats (67 females and 50 males) were equipped with a 5.4 g (4.1 ± 0.6% of bats body mass, cf. [54]) VHF transmitter designed at the Institute of Scientific Instruments of the Czech Academy of Sciences (ISI, Brno, Czech Republic), which was attached to the interscapular region using physiologically safe glue (ethyl 2-cyanoacrylate, Universum, Czech Republic) after trimming a patch of fur. The VHF transmitters were programmed to produce one pulse per 1.7 sec over an 18-day period (though they tended to fall off earlier) and had a range of approximately 2000 m. After release, the bats were continuously tracked using a four-station setup of the BAARA automatic radio tracking system (Biological AutomAted RAdiotelemetry system; for details see [55]), which took bearings every 2–10 minutes from all tagged bats simultaneously from sunset to sunrise. Bats were also located at foraging sites and day roosts manually, using two handheld two-element HB9CV and one six-element Yagi antennas (ISI, Brno, Czech Republic). The BAARA system utilises custom-made software (BAARAview version 1.7, Brno, Czech Republic) that converts position data (signal azimuth, strength and station position) directly to geographical coordinates.

Two types of data were used for spatial analysis, i) exact positions of a bat when spotted at close distance by a researcher [40] and, ii) positions estimated from manual bearings and using the four station BAARA system. When multiple stations took simultaneous bearings of a transmitter, its precise location was calculated by triangulation. Once converted to geographical coordinates, position data were imported into GIS software (ArcMap, [56]). Home ranges for 111 bats were estimated directly in BAARAView using standard minimum convex polygons (MCP; 95%) and core areas (CA; 50%) [57, 58]. To estimate radio tracking effort, we assessed the number of newly visited foraging sites for each bat-night. The probability of visiting a new site during a night was calculated as mean number of newly visited sites by all bats divided by the number of consecutive night (probability < 7% on the fifth bat-night; S1A and S1B Fig).

### Food availability

To estimate food availability at each foraging site, a 100 x 100 m area around the site was mapped and the number of date palms (*P*. *dactylifera*), and banana (*Musa sp*.), fig (*Ficus sp*.), mango (*Mangifera sp*.), citrus (*Citrus sp*.), jujube (*Ziziphus jujube*) and guava (*Psidium guajava*) trees with ripe fruit counted [40]. The assessment of food availability and fruit ripeness was continuous (during all days throughout radiotracking survey). Position data were continually visualized, so we had immediate information of the space-time distribution of all radio tracked bats. Whenever a new foraging site was found, the number of trees and fruit ripeness was evaluated. The foraging sites were then delineated using the GIS software. Because food availability was correlated with weather changes but not with reproductive cycle, data from two seasons are comparable.

### Genotyping and relatedness

Genomic DNA was extracted from the biopsy tissue of 63 individuals (38 females and 25 males, 31 from winter, 32 from spring) using the DNeasy® Blood & Tissue kit (QIAGEN Group) following the manufacturers protocol. Extraction of DNA from samples collected during summer was not possible due to sample degradation. Thirteen microsatellite loci were used for genotyping with fragments amplified via polymerase chain reaction under the same conditions as Hulva et al. [59] using fluorescently marked 66HDZ (80, 82, 105, 106, 110, 117, 304, 334, 341, 407, 413) and M3 (6, 121) primers [60, 61]. Fragment analysis was performed according to the protocol of Hulva et al. [59] on an ABI sequencer (Applied Biosystems), with allele sizes scored in GeneMapper (Applied Biosystems). Maximum likelihood estimation of pairwise relatedness (see [62]) was calculated in MLrelate [63].

### Network analysis

Undirected weighted networks were constructed for 63 fruit bats (31 from winter, 32 from spring) based on relatedness, with weight of the edges representative of the relatedness coefficient between individuals. For the same fruit bats, pair-wise foraging site sharing was quantified using GIS software, with a foraging site considered as shared when the positions of two fruit bats overlapped on the site during the same night. As it was not possible to observe interactions in the field, spatial co-occurrence was used as a proxy for foraging site sharing to construct networks [64]. Space sharing networks were constructed for both winter and spring, with the weight of the edges representing the percentage of shared foraging sites between two individuals. Filtered space sharing networks were also constructed using related pairs only to account for any disproportion in the number of related and unrelated pairs. Filtering social networks in this way can elucidate further hypotheses explaining network structure and may also highlight patterns that were predicted *a priori* [65]. In order to assess the potential role of co-roosting in social foraging, a co-roosting matrix was also constructed for 58 fruit bats. Exact positions of day roosts for the remaining 5 fruit bats were missing. Roost sharing between pairs of bats was defined as the number of days spent in the same roost. Network density (ρ) and node-based measures such as normalized degree (k), clustering coefficient (C) and weighted degree (s) were calculated from the space sharing networks. The network density is the number of edges in a network divided by the total possible edges. Density value of 1 mean that all fruit bats interacted with each other. The normalized degree (afterwards only degree) represents the number of edges connected to the node divided by all possible connections and captures individual gregariousness in terms of the number of interaction partners. The clustering coefficient is an unweighted measure of how well-connected the nodes are to their immediate neighbours [44]. Knowledge on clustering coefficient contributes to our understanding of how susceptible a population is to information flow [65]. The weighted degree combines the degree with the total weight of its edges, and it tells us about the sum of foraging sites shared by others. All networks were constructed and analysed in Gephi version 0.9.2 [66] and UCINET [67].

### Statistical analysis

The Kruskal-Wallis test was used to assess differences in MCP, CA, food availability, number of foraging sites visited, with season (winter, spring, summer) as the main factor. Bonferroni post-hoc tests were then used to compare the effects. The Kolmogorov-Smirnov test was used to compare pair-wise relatedness coefficient distribution between seasons (winter, spring). The multiple regression quadratic assignment procedure (MR-QAP) with double dekker semi-partialling was used to assess whether the space sharing network is predicted by relatedness or by co-roosting [68]. This method, an extension of the Mantel test, enables the dependent matrix to be regressed against one or more independent matrices and is generally used to model social relation (i.e. space sharing) using values of other relations such as genetic relatedness [67, 69]. Seasonal differences in degree, weighted degree and clustering coefficient were tested using T-test. Linear regression was used to test whether the number of sites visited predicted the degree. To generate appropriate significance levels according to our data structure, P values were generated using 10000 node-based permutations in all tests.

### Ethical statement

This study was carried out in strict accordance with the recommendations in the guidelines of the American Society of Mammalogists [70], which has been approved by the Institutional Animal Care and Use Committee of the Faculty of Science, Charles University in Prague. Capture and sampling of *R*. *aegyptiacus* were conducted under the permission granted by the Nature Conservation Egypt (# 22408921, 546223). Great care was taken during capturing, handling and sampling of bats to minimize stress and disturbance. The wound after this biopsy from wing membrane was fully closed and healed after 14 days [71]. Tagging of bats had no impact on movement of animals, or any other costs. Transmitters were programmed to transmit over 18 day but tended to fell of spontaneously earlier, leaving the skin intact.

## Results

Of the 117 bats radio tracked, 37 were followed during winter, 40 during spring and 40 during summer. In total, we obtained data from 927 bat-nights (8.35 ± 2.98 SD nights/bat), and 87609 locations (online dataset in Figshare).

**Fig 1:**
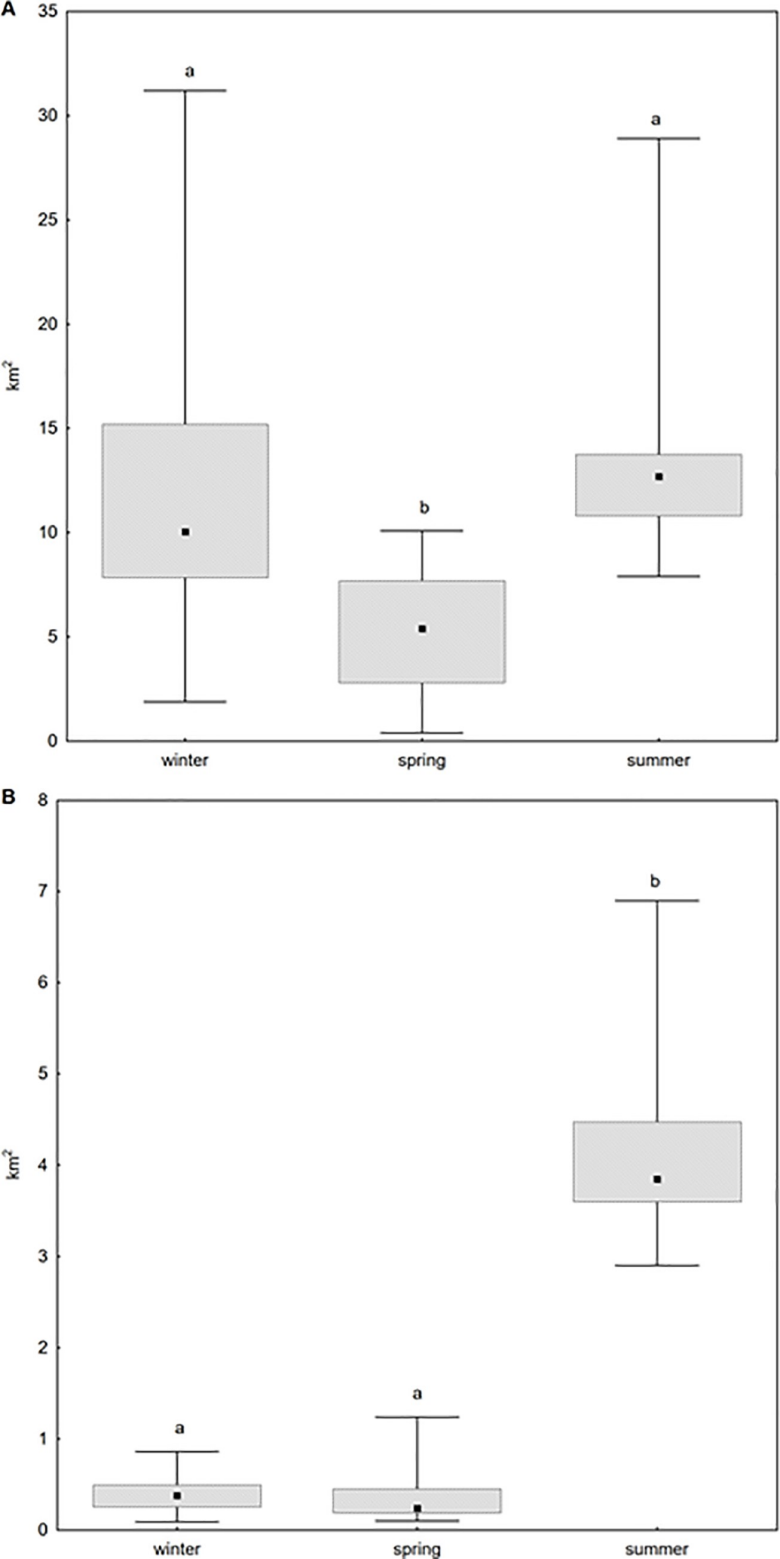
Home range (a) and core area (b) during three seasons. Boxes represent median ± quartiles (25%-75%), whiskers represent range; significance—ab, p < 0.001.

Seasonal differences in spatial activity characterised by home-range and core area size reflected food availability. The mean distance from the roost to the foraging site was 1485.2 ± 821.0 m. Season had significant effect on the size of MCP and CA. The lowest MCP was observed during spring (Kruskal-Wallis: H_2,111_ = 57.53; p<0.001; Fig 1A) and the CA was low during both winter and spring (H_2,111_ = 76.98; p<0.001; Fig 1B) compared to the summer. Food availability was the lowest in spring (H_2,42_ = 25.043; p < 0.001) when food was restricted to a single resource (dates). In comparison, up to seven alternative food resources were available in winter and summer, i.e. dates, mango, citrus, figs, jujube, guava and banana.

**Fig 2:**
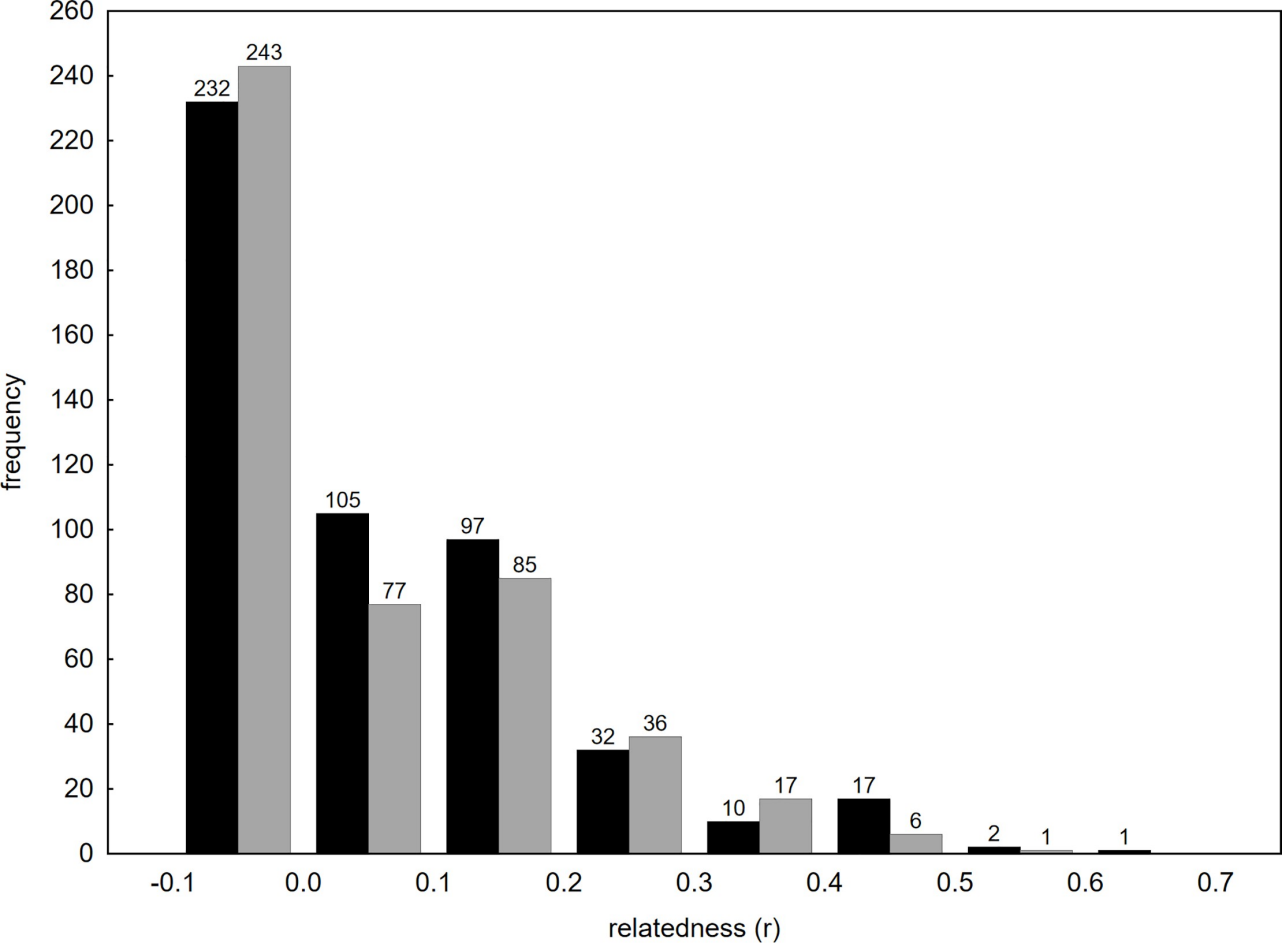
Distribution of pair-wise relatedness coefficients. Black columns represent winter, grey columns spring, the number above the columns is the count of observed values.

All thirteen microsatellite loci were polymorphic (mean number of alleles per locus = 6.15, ranging from 3 to 10). No difference was observed in the frequency distribution of pair-wise relatedness coefficients between seasons (Fig 2).

**Fig 3:**
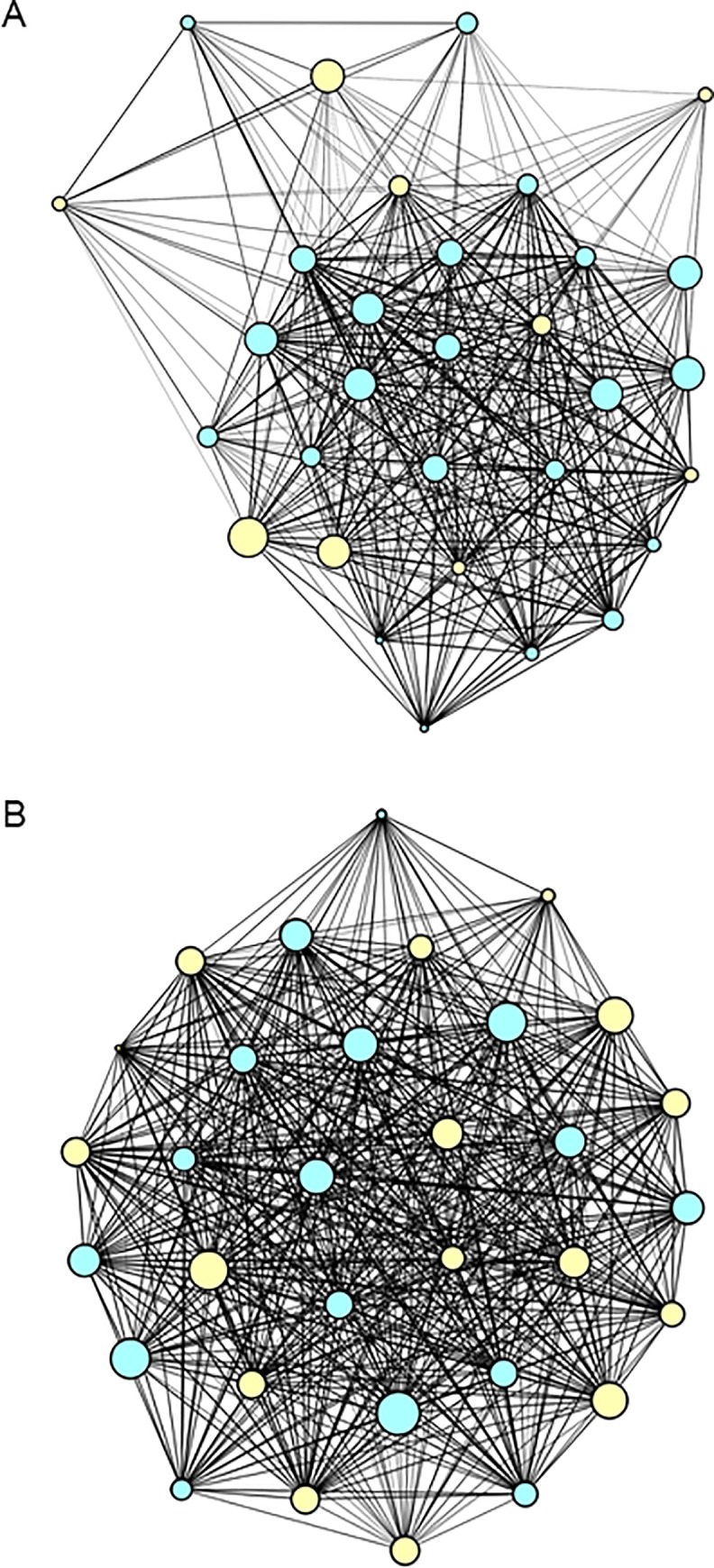
Space sharing networks. a) winter (n = 31), b) spring (n = 32); blue circles–females, yellow circles–males. Node size depends on the number of sites visited by an individual.

There was a significant correlation between the genetic and filtered space sharing network in winter (MR-QAP: r = 0.326; p < 0.001), but not in spring (r = -0.07628; p = 0.175). No link was found between the space sharing networks and co-roosting in winter (r = 0.14; p = 0.131), nor spring (r = 0.066; p = 0.290). Both, winter and spring space sharing networks (Fig 3A and 3B) had a high density (winter ρ = 0.867; spring ρ = 0.988).

**Fig 4:**
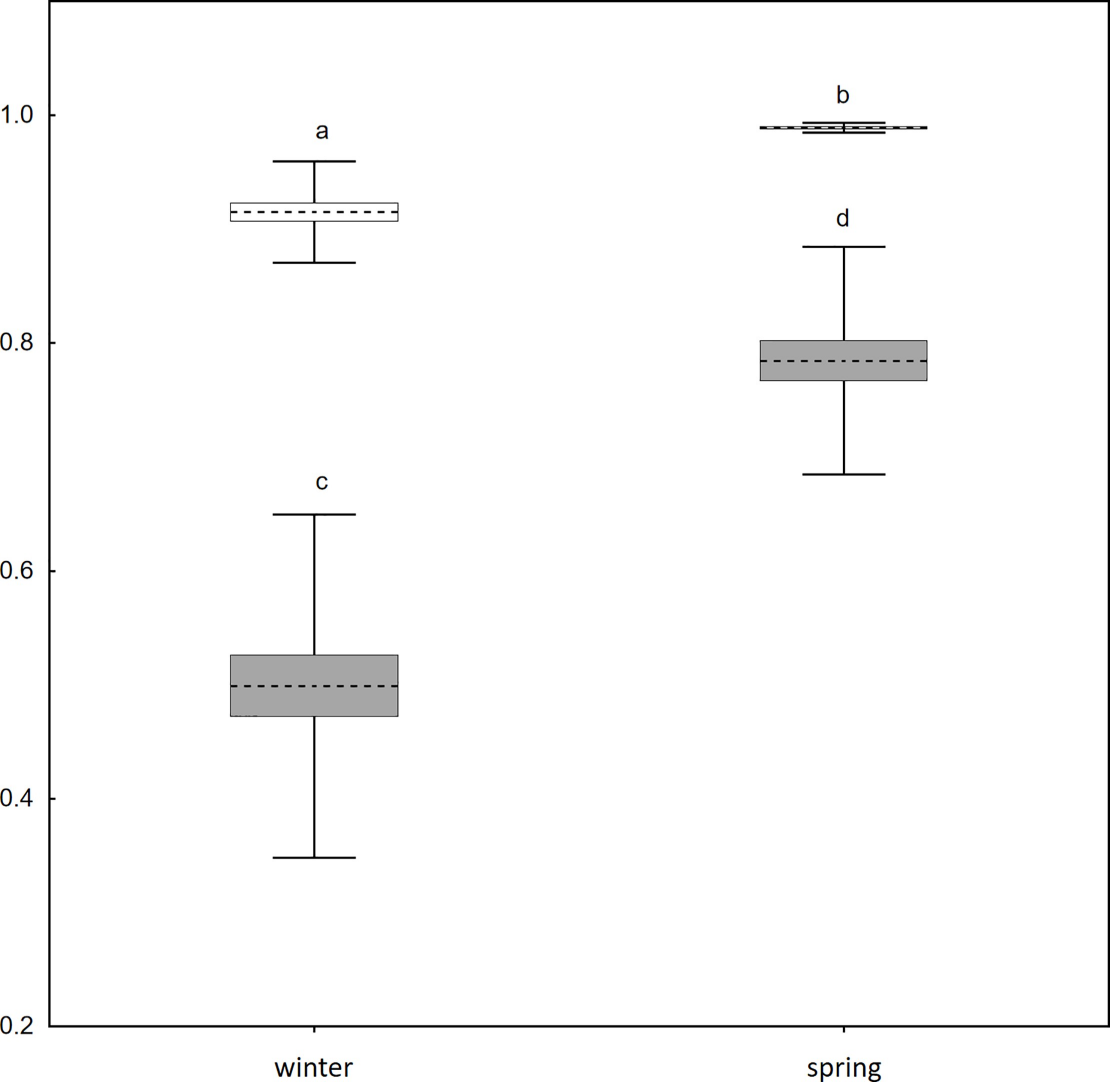
Seasonal difference in degree and clustering coefficient of space sharing networks. Boxes (grey–degree, clear–clustering coefficient) represent mean ± standard error; whiskers represent standard deviation; significance—ab,cd p < 0.001.

Season had a significant effect on both the degree (k_winter_ = 0.498; k_spring_ = 0.784; t = -8.885; p < 0.001; Fig 4) and the clustering coefficient (C_winter_ = 0.915; C_spring_ = 0.989; t = -9.377; p < 0.001; Fig 4). The weighted degree also differed significantly between seasons (s_winter_ = 14.967; s_spring_ = 24.314; t = -9,591; p < 0.001). The number of sites visited by an individual proved to be a predictor of its degree (adj. r^2^ = 0.301; F_1,61_ = 27.742; p < 0.001).

## Discussion

Our radio tracking data indicated profound seasonal differences in both food availability and Egyptian fruit bat spatial activity at the Dakhla oasis. We assume that larger MCPs during periods with high food availability reflect the presence of many small patches with ripe fruit scattered over the oasis. This may force the bats to switch between sites frequently and, consequently, forage over an extended area. Alternatively, fruit bats are not constrained by a single choice, thus they actively select different food sources. From the end of summer until spring, the bats relied mainly on harvestable fruit grown for export; indeed, frequent use of human-modified areas by Egyptian fruit bats has been previously reported in Mediterranean and desert environments [72]. During harvesting season, bats come to contact with people more often, which can also force them to move between patches to avoid conflict. At the Dakhla oasis, however, there were also marginal areas and abandoned palm plantations where fruit was never harvested, and some of the bats may have been foraging at these sites, resulting in large MCP. In spring, conditions became relatively harsh as fruit had either been harvested or new fruit had not yet ripened sufficiently. We expected home range size to increase when food became scarce; however, we observed the opposite trend. The small MCP and CA sizes observed during times of poor food availability may have been caused by i) few suitable foraging sites being situated relatively close to each other, and ii) the high costs of foraging over longer distances under harsh conditions at a time when females are lactating and have high energy demands [38]. The relationships observed in our study were inconsistent with the well-established negative relationship between home range size and local food availability previously noted for a wide variety of other species [73–75].

Our data revealed a significant correlation between the kinship and the filtered space sharing network in winter, but not in spring. This could have resulted from differences in food distribution over the two seasons, with food being scattered all over the oasis in winter but reduced to a few foraging sites in the eastern part of the oasis in spring. Therefore, in spring, all animals foraged together and shared sites where food was still available, while in winter, the bats had the opportunity to select a food source and we propose this selection to be partially kin-biased. The positive link between relatedness and foraging during winter may also reflect reproductive cycle. In such cases, foraging with kin may be beneficial in relation to the costs of finding a good site and ease of access to information [33, 50, 51]. Spatial association patterns may also depend strongly on roosting biology [31, 50] and roost-based exchange of information about foraging sites which has been previously reported in bats by Wilkinson [76] and Kerth and Reckardt [77]. However, in our study, co-roosting was shown to have no effect on foraging space sharing similarly as shown in Bechstein’s bats (*Myotis bechsteinii*, [31]).

Both, winter and spring space sharing networks had relatively high connectivity; however, the degree and the clustering coefficient was significantly higher in spring. Our results also showed a significantly higher weighed degree in spring, suggesting that, in season with scarce food offer, fruit bats not only interact with more individuals, but also share higher proportion of sites with each other. Individuals laying further from the network centre visited fewer foraging sites and had lower degree, meaning they were less gregarious. In each case, the patterns of space use observed are likely to be related to the abundance and spatial distribution of resources, i.e. food [78]. As the food sources went scarce, fruit bats tended to share few small sites where fruit was still available, resulting in group cohesion and more connected network. Overall, the high connectivity of space sharing networks may be explained also by communal knowledge about foraging sites.

Although we have no evidence of information flow, we might expect information spread to follow the pattern of space sharing [79, 80]. We assumed, that individuals that share a foraging site were more likely to exchange information [81]. Transmission of information tends to be more rapid and easier in dense networks where each node has high degree [65, 82]. An example provided by the innovative foraging techniques observed in Japanese macaques (*Macaca fuscata*), where it is reasonable to assume that degree in such innovators determines how rapidly the innovation will spread [65, 83]. Information spread is also encouraged by a high mean clustering coefficient [84]; indeed, in a previous study on Spix’s disk-winged bat (*Thyroptera tricolor*), Chaverri [45] reported a higher social network clustering coefficient (C ≥ 0.86) than that reported for other mammals [85–90], suggesting that many social networks may be less interconnected than those of bats.

Fruit bats that visited more foraging sites tended to have a higher degree, which would appear logical, as more explorative individuals have more chances to associate with other individuals [91]. In this way, we propose that fruit bats with less knowledge would benefit from associating with such individuals, as it would help them acquire information about new foraging sites e.g. via local enhancement [92] or producer–scrounger interactions [93]. However, as our data from few GPS-tagged fruit bats indicate, following behaviour cannot be excluded either (Bachorec et al. in prep.).

Though there are numerous studies reporting non-kin foraging associations in bats (e.g. in Pallas’s mastiff bat (*Molossus molossus*) [94]; the greater mouse-tailed bat (*Rhinopoma microphyllum*) [95]; and the greater spear-nosed bat (*Phyllostomus hastatus*) [15], our findings show that Egyptian fruit bats choose to forage with more related individuals but only during periods with plentiful food sources.

In this study, we demonstrated that seasonal changes in food availability have a marked effect on fruit bat foraging behaviour. When fruit was scarce (spring), whether due to seasonality or harvesting, spatial analysis showed a decrease in both MCP and CA, suggesting a lack of food patches. Seasonal differences in food distribution also had an impact on the connectivity of space sharing networks, with abundant food sources in winter leading to a decrease in network connectivity and kin-biased site sharing. On the other hand, fewer sources in spring led to group cohesion and un-biased site sharing. Therefore, in harsh conditions, individuals would benefit from following the group decisions, while in good conditions, individuals may rely on foraging decisions of relatives. Overall, coherent spatial distribution of fruit bats seems to be beneficial in agricultural areas with predictable harvesting periods and seasonal fluctuations in food availability.

## Supporting information

S1 Fig Radiotracking effort. a) new foraging sites visited per night; b) probability of visiting a new site.
(PDF)

S2 Fig Genetic networks. a) winter; b) spring. Males are depicted by triangles, females by squares. Node size depends on the degree of an individual.
(PDF)
