# Supplementary figures and images for: Spatial networks differ when food supply changes: Foraging strategy of Egyptian fruit bats
Source: PLoS One. 2020 Feb 25;15(2):e0229110. doi: 10.1371/journal.pone.0229110 (PMC7041839; doi:10.1371/journal.pone.0229110)

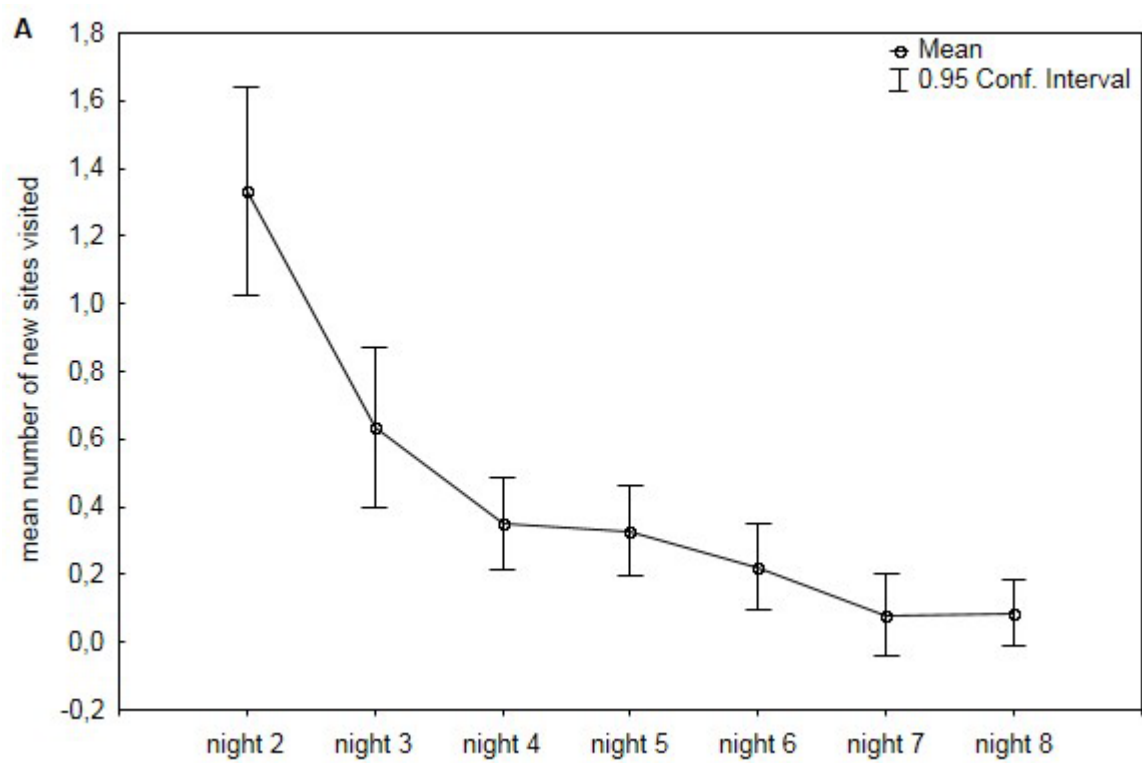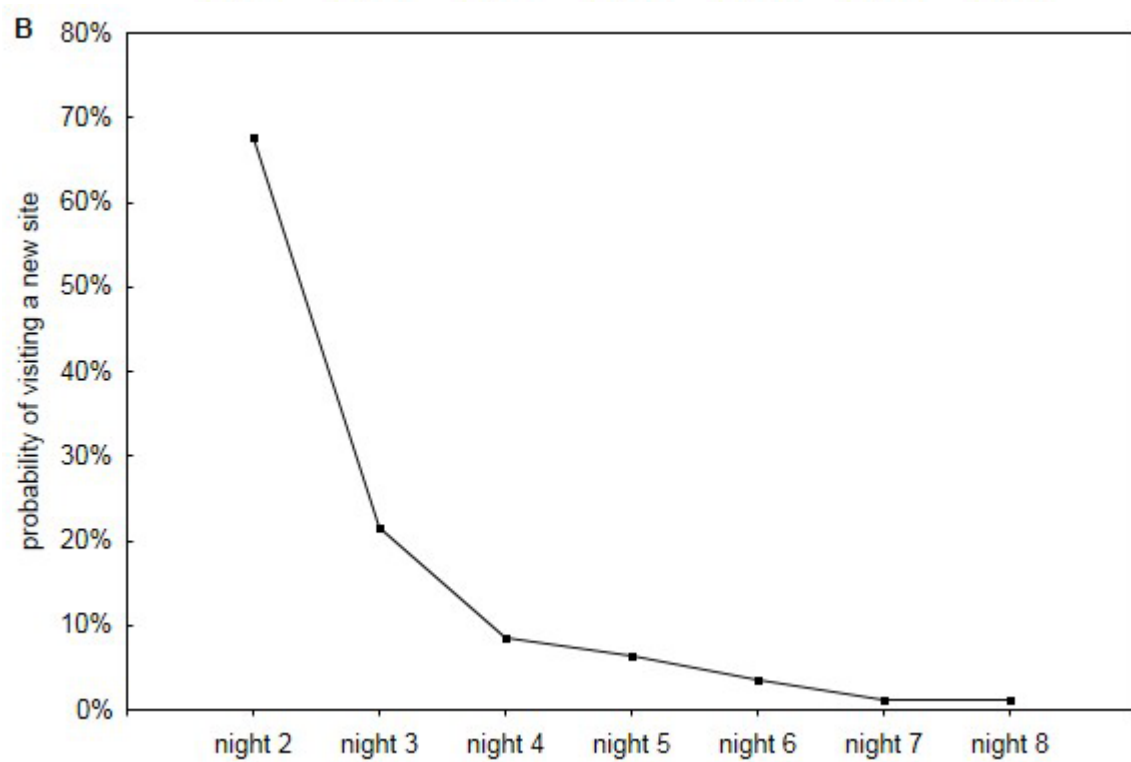

Supplement: S1 Fig — a) new foraging sites visited per night; b) probability of visiting a new site. (PDF) [file pone.0229110.s001.pdf]

A

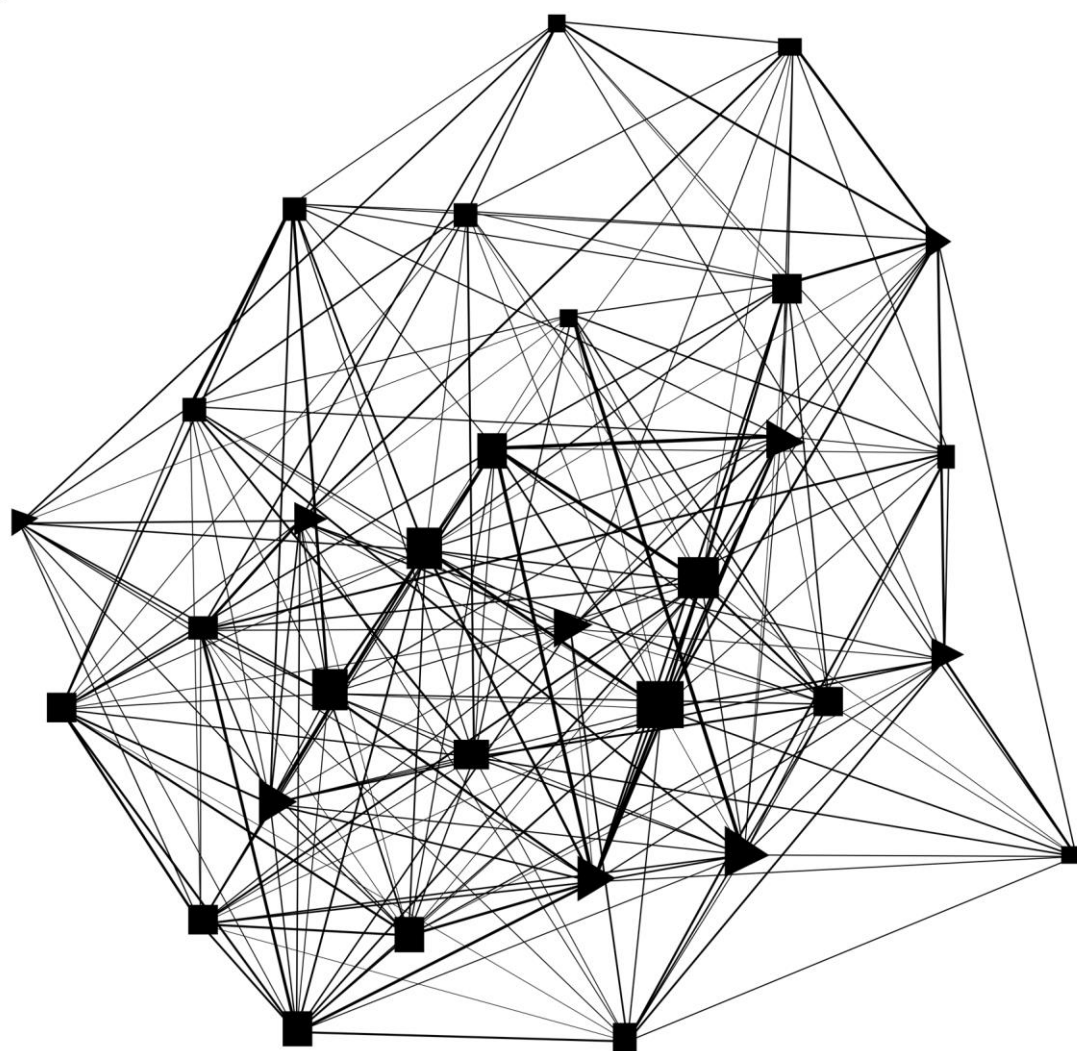

**B**

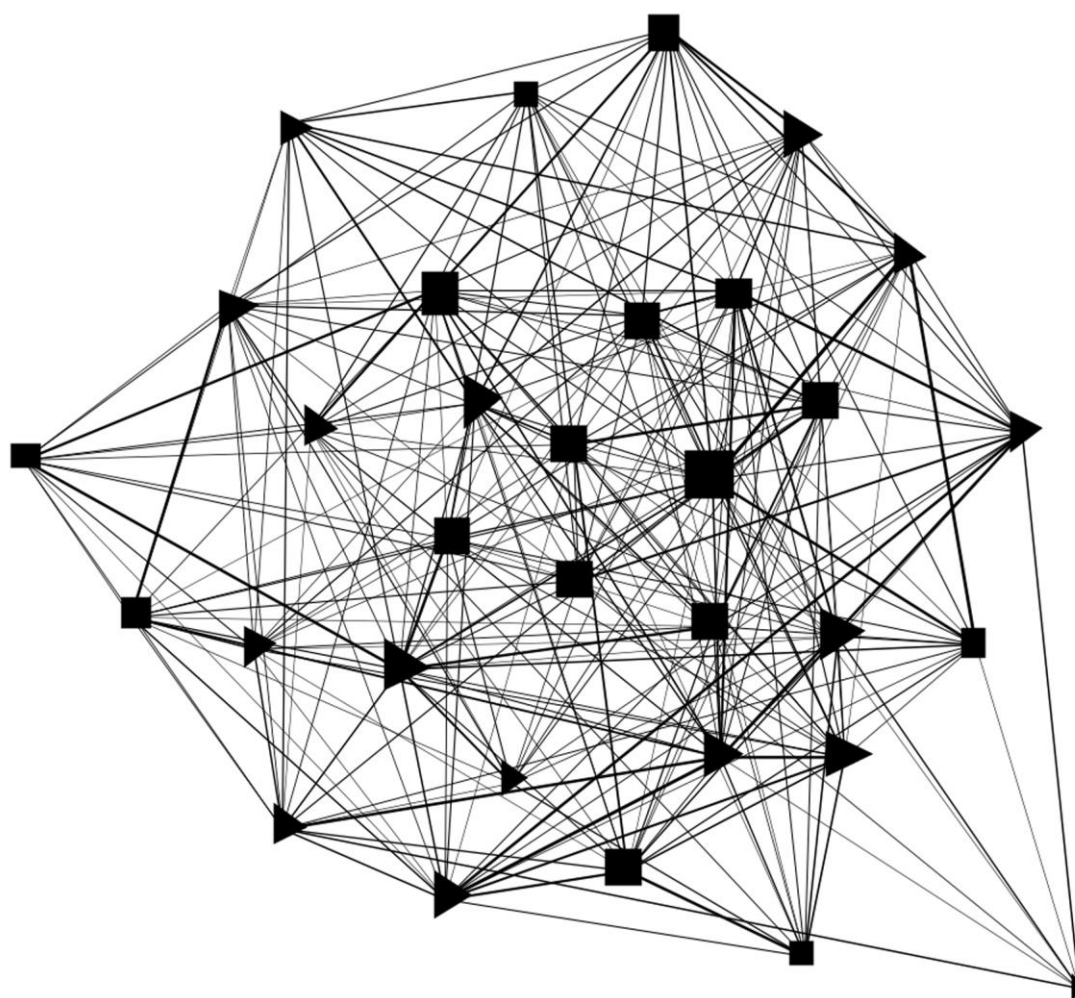

Supplement: S2 Fig — a) winter; b) spring. Males are depicted by triangles, females by squares. Node size depends on the degree of an individual. (PDF) [file pone.0229110.s002.pdf]
